# Gene transcription and steviol glycoside accumulation in *Stevia rebaudiana* under polyethylene glycol‐induced drought stress in greenhouse cultivation

**DOI:** 10.1002/2211-5463.12099

**Published:** 2016-08-30

**Authors:** Shokoofeh Hajihashemi, Jan M. C. Geuns

**Affiliations:** ^1^ Plant Biology DepartmentFaculty of ScienceBehbahan Khatam Alanbia University of TechnologyKhuzestanIran; ^2^ Laboratory of Functional BiologyKULeuvenHeverlee‐LeuvenBelgium

**Keywords:** HPLC, polyethylene glycol, real‐time quantitative PCR, *Stevia rebaudiana*, steviol glycosides

## Abstract

*Stevia rebaudiana* is a sweet herb of the Astraceae family, which is cultivated for the natural sweeteners it contains. The aim of this study was to assess the effect of drought, simulated by the application of polyethylene glycol (5%, 10%, and 15% w/v), on the content of steviol glycosides (SVglys) and transcription levels of six genes involved in the biosynthesis of these natural sweeteners. The transcription levels of *ent‐kaurene synthase*,* ent‐kaurene oxidase*,* ent‐kaurenoic acid hydroxylase*, and three UDP‐dependent glycosyltransferases, *UGT85C2*,*UGT74G1* and *UGT76G1* were downregulated under polyethylene glycol treatment. Polyethylene glycol treatment significantly decreased the amount of stevioside, rebaudioside A, B, C and F, steviolbioside, dulcoside A, rubusoside, and total SVglys. These results strongly suggest a close relationship of SVglys content with the transcription of genes involved in the SVglys biosynthesis pathway. Comparing the observations of the present study with other reports provided the knowledge that the *Stevia* response to drought stress can be influenced by different environmental and experimental factors, in addition to intensity of drought stress. In conclusion, these results strongly suggest that polyethylene glycol‐induced drought stress has a negative effect on the content of SVglys and transcription of SVglys biosynthetic genes and that this should be investigated further. We recommend that sufficient irrigation of *Stevia* is required to obtain a high content of SVglys.

**Abbreviations**
AcCN: acetonitrile
CPPS: copalyl diphosphate synthase
Dul: dulcoside
GA: gibberellic acid
GGDP: geranylgeranyl‐diphosphate
GTs: glycosyltransferases
KAH: kaurenoic acid hydroxylase
KO: kaurene oxidase
KS: kaurene synthase
LDM: leaf dry mass
Reb: rebaudioside
RT‐qPCR: real‐time quantitative polymerase chain reaction
Rub: rubusoside
SB: steviolbioside
ST: stevioside
SVglys: steviol glycosides
SV: steviol

*Stevia rebaudiana* Bertoni is a perennial herb belonging to the Asteraceae family. It is a natural sweetener plant known as “Sweet Weed”, “Sweet Leaf”, “Sweet Herb”, and “Honey Leaf”, which is estimated to be 30 times sweeter than cane sugar. Leaves of *S. rebaudiana* accumulate up to 30% of steviol glycosides (SVglys), the compounds responsible for its sweet taste 1. SVglys are used as a calorie‐free sweetener in many countries of the world including China, Japan, Korea, Australia, New Zealand, and many countries of the European Union. *Stevia* is a medicinal plant with a huge demand in pharmaceutical, food and beverage industries as a source of low‐calorie and high‐potency natural sweeteners. Many studies showed that SVglys have different beneficial pharmacological effects, such as lowering of blood pressure, regulation of blood glucose in type 2 diabetes while enhancing insulin sensitivity, prevention of atherosclerosis, prevention of certain forms of cancers and as a free radical scavenger 2, 3, 4.

SVglys are glycosylated derivatives of the diterpenoid steviol. The major SVglys in *S. rebaudiana* are stevioside (ST) and rebaudioside A (Reb A). Other SVglys of steviolbioside (SB), Reb B, C, D, E, F and dulcoside A (Dul A) are present in smaller amounts 5, 6, 7. In *Stevia*, geranylgeranyl‐diphosphate (GGDP) is converted into steviol by the consecutive action of four enzymes: copalyl diphosphate synthase (CPPS), ent‐kaurene synthase (KS), ent‐kaurene oxidase (KO), and ent‐kaurenoic acid hydroxylase (KAH). Different SVglys are formed by glycosylation of steviol by specific glycosyltransferases (UGTs) 8, 9, 10, 11 (Fig. 2).

Plants frequently encounter unfavorable growth conditions. They are challenged by stressful environmental conditions like water deficit, which leads to changes in molecular, biochemical, and physiological processes. Consequently, plant growth and development are adversely affected 12, 13. The responses are regulated by multiple signaling pathways which result in changes in the patterns of gene expression. *Stevia rebaudiana* originates from Paraguay, a semihumid area with a yearly average rainfall of 1500 mm. Now cultivation of *Stevia* has spread to other regions of the world, including Canada and some regions of Asia and Europe. One critical point in *Stevia* cultivation is sufficient irrigation to obtain greater profits. According to the finding of Hajihashemi and Ehsanpour 14, *S. rebaudiana* showed little resistance to *in vitro* polyethylene glycol‐induced drought stress under controlled conditions. Polyethylene glycol negatively affected plant growth, photosynthetic pigments, proteins, enzymes 14, 15. Also polyethylene glycol‐induced drought stress under *in vitro* condition negatively affected the transcription of *ent‐KO*,* UGT85C2* and *UGT76G1* genes 16. Our previous study was carried out under *in vitro* condition which is an artificial medium supplemented with sucrose. Therefore, it was imperative to know how *Stevia* reacts to polyethylene glycol treatment under controlled greenhouse conditions which are close to field culture. Understanding how plants respond to drought stress can play an important role in improving *Stevia* management and performance, especially as the climate‐change scenarios suggest an increase in aridity in many areas of the globe.

To make the first step toward the production of drought tolerant *Stevia* plants, the expression of some related genes should be analyzed under drought stress. Polyethylene glycol treatment under greenhouse culture provide us compelling evidence to heighten awareness of the mechanism by which the vital step of SVglys biosynthesis is regulated by drought stress and its correlation with gene transcription. The objective of this study was to (a) measure SVglys content (e.g., ST, Reb A, B, C, F, SB, Dul A, Rub, and total SVglys) and (b) distinguish the transcription of *ent‐KS*,* ent‐KO*,* ent‐KAH*,* UGT85C2*,* UGT74G1*, and *UGT76G1* genes to understand the correlation between genes transcription and SVglys accumulation in polyethylene glycol‐treated *S. rebaudiana*.

## Materials and methods

The *S. rebaudiana* plants were provided by the Laboratory of Functional Biology, KULeuven University, Leuven, Belgium. *Stevia* propagation was carried out by tissue culture. The *in vitro* propagated plants were grown to the seedling stage under normal greenhouse conditions with supplementary light to extend the photoperiod to 16 h per day. Air temperature ranged from 22 °C to 26 °C during the day and 15 °C to 18 °C during the night. Humidity range was between 40% and 60%. Two‐month old plants of similar size were transferred to 1.5 L pots filled with perlite and vermiculite. The plants were irrigated every other day and fertilized once a week by nutrient solution (pH 6.0). The 6‐month old plants were irrigated with 0% (distilled water), 5%, 10%, and 15% w/v polyethylene glycol (molecular weight 6000; −0.08, −0.49, −1.4, −2.9 MPa, respectively) solutions. Polyethylene glycol treatment continued for 1 month. All plants were harvested 2 days after the last day of polyethylene glycol treatment to be used for further analysis. All plant samples were immediately frozen in liquid nitrogen and stored at −80 °C until further use. Some of frozen leaves were freeze‐dried for SVglys analysis. The experiment was designed as a completely randomized block. Each experiment was repeated three times at different periods. Each experiment consisted of six replicates per treatment.

### Water extraction of leaves

Twenty mg of freeze‐dried and pulverized leaves were extracted three times consecutively by boiling, each sample three times for 15 min in 300 μL HPLC quality water. The extracts were mixed, pooled, centrifuged, and filtered to remove particles. To avoid possible losses, no purification step was used. The extracts were stored at 4 °C for further use.

### SVglys analysis

To measure the SVglys, 20 μL of extract was injected in the HPLC with two Grace Alltima C_18_ columns in series (250 × 4.6 mm ID, 5 μm particle size; UV detection was at 200 nm, 10 mm path length). The HPLC system consisted of two LC‐20AT pumps and a SIL‐HTc autosampler from Shimadzu, Deurne, Belgium. The solvent flow rate was 1.0 mL·min^−1^ and the gradient of acetonitrile (AcCN): 1.0 mm phosphoric acid was as follows: *t* 0 min: 34% AcCN (v/v), 4 min: 35%, 10 min: 42%, 16 min: 42%, 16.1–25 min: 34%, 25 min: stop 17. The SVglys measured were: ST, Reb A, B, C, F, Dul A, Rub, and SB. For quantification, Reb A (purity > 99%) was used as an external standard 2. Results were expressed as amount of SVgly % of leaf dry mass (LDM).

### Gene transcription analysis using RT‐qPCR

The frozen leaves were used to extract total RNA using the Plant RNA Mini Kit (Thermo Fisher Scientific, UK), according to the manufacturer's instructions. The amount of 2.3 μg of total RNA was reversely transcribed with 50 U AMV‐reverse transcriptase in a total volume of 25 μL of Master Mix, for cDNA synthesis. Master mix was a mixture of 2.5 μm oligo (dT)_16_ primer, 20 U RNase inhibitor, 1× PCR buffer, 5 mm MgCl_2_, and 1 mm of each dNTP (GeneAmp RNA PCR kit; Roche Molecular Systems, Branchburg, NJ, USA). The house‐keeping genes of *β‐Actin* and *18S rRNA* were used in this experiment. OligoCalc software was used to design primers for both target and house‐keeping genes 18 (Table 1).

**Table 1:** List of primers used in RT‐qPCR and house‐keeping genes. Kaurene synthase (*ent‐KS*), kaurene oxidase (*ent‐KO*), kaurenoic acid hydroxylase (*ent‐KAH*), UDP‐dependent glycosyltransferases of *UGT85C2*,* UGT74G1*, and *UGT76G1*

Gene	Primer sequence 5′→3′ (forward/reverse)	Amplicon length (bp)	Accession number
*ent‐KS1‐1*	GCTCTGATTGAACACACGATTATC/ TCCTATGTAGAGTGAATCTAAGAGG	151	AF097310
*ent‐KO*	GCTGTGATGAAGTCTCTTATTAAA/ CCATAGTGGTGTCTGATGATTCAAT	162	AY364317
*ent‐KAH*	CCATATTCACCATCCGACTTGG/ GGGTAGTGAAGATCTCCTTAGC	151	Brandle and Richman 20
*UGT85C2*	TCGATGAGTTGGAGCCTAGTATT/ CTAAACTGTATCCATGGAGACTC	153	AY345978
*UGT74G1*	TGCATGAACTGGTTAGACGATAAG/ GCATCCTACTGATTCGTGTGCTA	274	AY345982
*UGT76G1*	GCAGCTTACTAGACCACGATC/ CTCATCCACTTCACTAGTACTAC	107	AY345974
*18S rRNA* a	CCGGCGACGCATCATT/ AGGCCACTATCCTACCATCGAA	59	–
*β‐Actin*	AGCAACTGGGATGACATGGAA/ GGAGCGACACGAAGTTCATTG	65	AF548026

a Using specific primers designed for *18S rRNA*, a segment of 400 bp of *Stevia rebaudiana* was obtained and used as a template to design primers convenient for RT‐qPCR.

SYBR green binds to double‐stranded DNA and the amplification products emit fluorescence. Real‐time quantitative PCR based on the emitted fluorescence, to quantify the target transcript accumulation relative to the house‐keeping gene transcript. A 96‐well plate was used to carry out the SYBR green one‐step RT‐qPCR. The PCR reaction was containing 2.5 μL RNase‐free water, 0.2 μL of each primer (10 μmol·L^−1^), 5 μL SYBR green mix, 0.1 μL CXR and 2 μL of cDNA (5 ng·μL^−1^), in a total volume of 10 μL. The reactions were run in triplicate in an ABI prism 7000 sequence detection system (ABI Prism 7000 SDS; Thermo Fisher Scientific). The thermal cycles of 95 °C for 20 s, followed by 40 cycles of 95 °C for 3 s and 62 °C for 30 s. Melting curve (dissociation) analysis (60–95 °C) was done to verify amplicon specificity after 40 cycles at 95 °C for 15 s, 62 °C for 1 min and 95 °C for 15 s. *β‐Actin* in addition to *18S rRNA* were used to normalize the data individually. According to the 2^−ΔΔCT^ method, the abundance of targeted gene transcripts was set relative to control plants (with no treatment) 19. step one software (version 2.1; Thermo Fisher Scientific) was used to analyze the data of RT‐qPCR.

### Statistical analysis

A Randomized Complete Block Design was used for all experiments with three biological and technical replications. The Duncan test's spss (version 16; SPSS, New York, NY, USA) statistical package was applied to calculate the significant differences (at the 5% level) between means. The results of statistical analysis are shown by superscripted letters after the numbers in tables to reveal significant differences.

## Results

**Figure 1:**
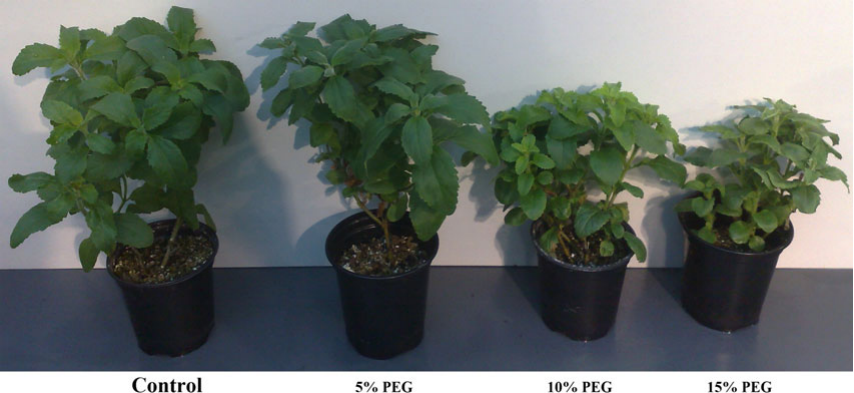
*Stevia rebaudiana* plants treated with different concentrations of poly(ethylene glycol) (molecular weight of 6000) at 0%, 5%, 10%, and 15% w/v.

Figure 1 shows the *Stevia* plants under different treatments of polyethylene glycol. The plants were treated with 0%, 5%, 10%, and 15% of polyethylene glycol which resulted in smaller plants. The SVglys content of leaves was analyzed in the treated plants which are shown in Table 2. The accumulation of SVglys in leaves of *Stevia* was negatively influenced by poly(ethylene glycol) treatment. Polyethylene glycol treatment significantly decreased the SVglys content, which was most pronounced at 15% of polyethylene glycol. Polyethylene glycol treatments significantly decreased ST, Reb A, Reb B, C, F, Dul A, Rub, and SB. The smallest and greatest contents of ST, Reb A, Reb B, C, F, Dul A, Rub, and SB were observed at 0% and 15% of polyethylene glycol, respectively. ST and Reb A are two important SVglys and *S. rebaudiana* has a strong tendency to accumulate a large share of its SVglys as ST, about three times more than Reb A in control plant (Reb A/ST ratio about 0.39). Different levels of polyethylene glycol at 5%, 10%, and 15% decreased ST content by about 18%, 25%, and 52%, respectively. Reb A content significantly decreased by about 28%, 39%, and 48% at 5%, 10%, and 15% of polyethylene glycol treatment, respectively. Polyethylene glycol treatment had a significant and similar negative effect on both ST and Reb A contents and therefore no significant changes were observed in Reb A/ST ratio under different levels of polyethylene glycol. The total amount of SVglys in the leaves of control plant was about 8.3% of LDM. In polyethylene glycol‐treated plants, the total SVglys content reduced by about 21%, 32%, and 53% at 5%, 10%, and 15% of polyethylene glycol, respectively.

**Table 2:** SVglys content (% LDM) in leaves of *S. rebaudiana* treated with polyethylene glycol (mean ± SD). Values are expressed as means of three independent experiments. Dulcoside A (Dul A), steviolbioside (SB), stevioside (ST), rebaudioside A (Reb A), Reb B, C and D, rubusoside (Rub). Treatments with the same lower‐case letters were not significantly different based on mean comparison by Duncan's test at *P* ˂ 0.05

Polyethylene glycol treatment				
Svgly%	Control	5%	10%	15%
RebA	1.957 ± 0.167^a^	1.399 ± 0.163^b^	1.196 ± 0.101^bc^	1.006 ± 0.075^d^
ST	5.057 ± 0.355^a^	4.147 ± 0.207^b^	3.771 ± 0.164^c^	2.438 ± 0.127^d^
Reb F	0.346 ± 0.056^a^	0.281 ± 0.038^b^	0.136 ± 0.015^c^	0.070 ± 0.008^d^
Reb C	0.447 ± 0.033^a^	0.356 ± 0.030^b^	0.272 ± 0.018^c^	0.239 ± 0.010^d^
Dulc A	0.291 ± 0.040^a^	0.214 ± 0.026^b^	0.127 ± 0.016^c^	0.065 ± 0.004^d^
Rub	0.086 ± 0.010^a^	0.065 ± 0.008^b^	0.044 ± 0.004^c^	0.028 ± 0.005^d^
Reb B	0.058 ± 0.003^a^	0.040 ± 0.003^b^	0.030 ± 0.004^c^	0.023 ± 0.001^d^
SB	0.021 ± 0.001^a^	0.015 ± 0.001^b^	0.011 ± 0.001^c^	0.005 ± 0.000^d^
Total	8.263 ± 0.434^a^	6.517 ± 0.376^b^	5.587 ± 0.239^c^	3.874 ± 0.220^d^

According to results from SVglys analysis and some other preliminary experiments (data not shown), we decided to do the RT‐qPCR analysis on plants treated with 10% of polyethylene glycol. The house‐keeping genes of *β‐Actin* or *18S rRNA* were used to normalize the results of RT‐qPCR. The transcription pattern of both house‐keeping genes was similar. Samples had a good purity because no signal amplification was observed in the negative control sample. The values were reported relative to *β‐Actin* because the expression patterns of samples relative to *β‐Actin* or *18S rRNA* were almost similar. Also, the melting curves of all samples were the same.

**Figure 2:**
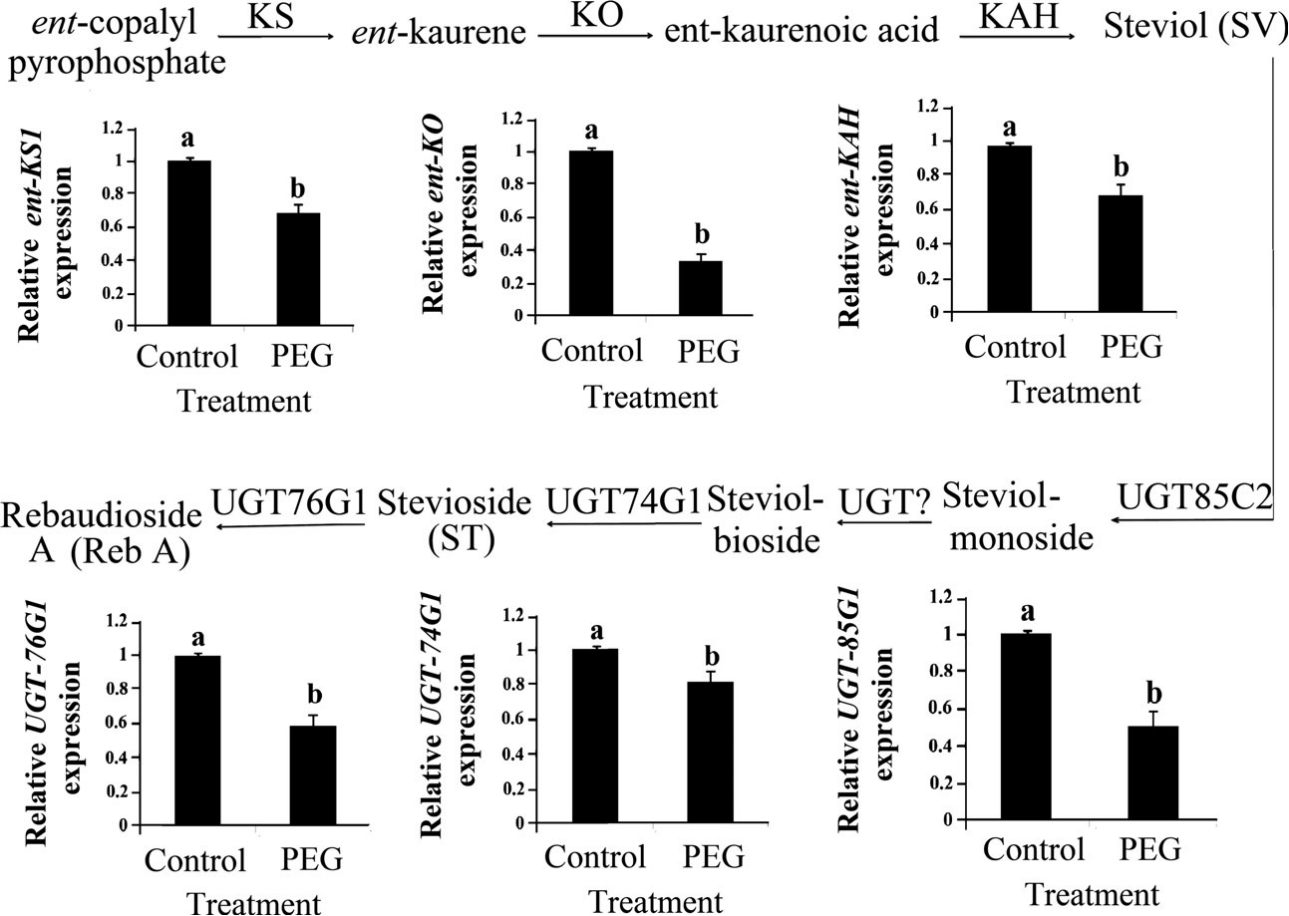
The biosynthetic pathway of steviol glycosides. The transcription of *ent‐KS1*,* ent‐KO*,* ent‐KAH*,*UGT85C2, UGT74G1*, and *UGT76G1* of *S. rebaudiana* involved in the SVglys biosynthesis, relative to that of *β‐Actin* in plants subjected to polyethylene glycol treatments. Kaurene synthase (KS), Kaurene oxidase (KO), Kaurenoic acid hydroxylase (KAH), UDP‐dependent glycosyltransfrases(UGT). Treatments with the same lower‐case letters were not significantly different based on mean comparison by Duncan's test at *P* ˂ 0.05.

Figure 2 shows the transcription patterns of investigated genes in plants subjected to polyethylene glycol treatment. A significant reduction was observed in the transcription of *ent‐KS1*,* ent‐KO*, and *ent–KAH* genes under polyethylene glycol treatment by about 31%, 67%, and 30%, respectively (Fig. 2). The *UGT74G1*,* UGT76G1*, and *UGT85C* transcription significantly decreased in polyethylene glycol‐treated plants by about 19%, 41%, and 50%, respectively (Fig. 2). *Ent‐KO* showed the greatest decrease in gene transcription among the six investigated genes. In addition, the adverse effect of polyethylene glycol on the transcription of three genes (*ent‐KO*,* UGT85C2*, and *UGT76G1*) was more than that of the other three genes (*ent‐KS1*,* ent–KAH*, and *UGT74G1*).

## Discussion

**Figure 3:**
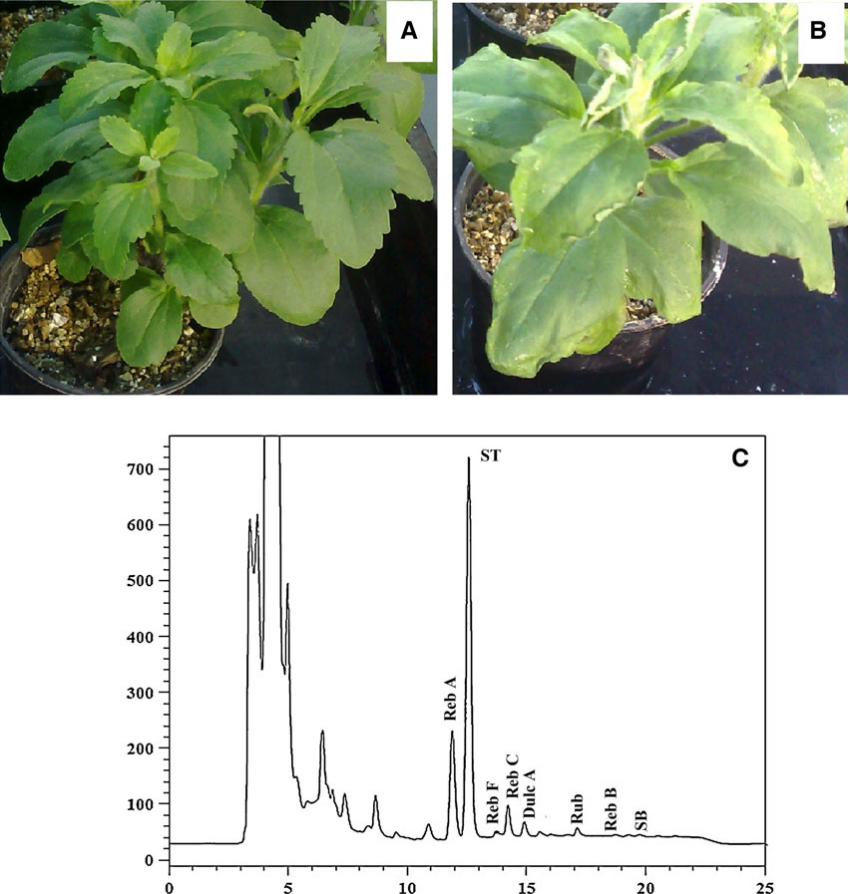
Photos of *S. rebaudiana* plants taken 2 days after a 1‐month treatment with polyethylene glycol (PEG0% and 10% w/v, respectively, panels A and B). HPLC trace of *S. rebaudiana* leaf extracts (C).

*Stevia rebaudiana* is a source of new sweeteners with a huge economic potential because of its SVglys accumulation (Fig. 3). Therefore, it is a critical point to study the elements which may negatively affect SVglys accumulation. Plant growth and secondary metabolism are commonly regulated by external cues such as light, temperature, and water availability 21, 22. Drought stress is one of the critical limiting factors, adversely affecting *Stevia* plant growth and performance 14, 15, 16, 23. Evidently, the negative effect of polyethylene glycol on plant growth was observed at high level of polyethylene glycol in the present study (Fig. 3). In this study, the changes of SVglys contents and the transcript levels of some corresponding biosynthetic genes were investigated under different polyethylene glycol treatment. The discovery of reduction in SVglys contents in polyethylene glycol‐treated plants can be correlated with the downregulation of the transcription of *ent‐KS1*,* ent‐KO*,* ent–KAH*,* UGT74G1*,* UGT76G1*, and *UGT85C2* genes, which are involved in SVglys biosynthesis pathway. The inhibition of *ent‐KS1* and *ent‐KO* transcription can reduce *ent*‐Kauronic acid which is the precursor of SVglys. According to Kumar *et al*. 24, a positive correlation can be detected between the transcription of *ent*‐KO transcription and SVglys content, which is supported by the results from the present study. The reduction in plant height under polyethylene glycol treatment can be contributed to the downregulated transcription of *ent‐KS* and *ent‐KO* genes because ent‐KS and ent‐KO enzymes catalyze gibberellin biosynthesis (Fig. 1). Paclobutrazol treatment decreases plant growth via blocking GA biosynthesis, more specifically at the step of *ent*‐kaurene oxidation 25, 26. It is known that under *in vitro* condition 16, *ent‐KO* transcription significantly decreased in the polyethylene glycol‐treated plants while no changes were observed in the transcription of *ent‐KS1*. Yang *et al*. 27 reported that the transcription of *ent‐KS1* and *ent‐KO* did not change under drought stress. Another conflict was observed about the transcription of *ent‐KAH*. Polyethylene glycol treatment under *in vitro* culture had no significant effect on the transcription of *ent‐KAH* 16, but in the present study, a significant reduction was observed in it.

Glycosyltransferases catalyze the transfer of a glycosyl moiety from an activated donor to an acceptor molecule, forming a glycosidic bond 28. In *S. rebaudiana*, UGTs trigger the downstream steps in the synthesis of steviol glycosides 1. The transcription of *UGT85C2* and *UGT76G1* genes showed a significant reduction in polyethylene glycol treatment which is supported by other reports 16, 27. UGT85C2 enzyme forms steviolmonoside by adding a C‐13 glucose to steviol 18. Thus, the significant correlation between total SVgly accumulation and transcription of *UGT85C2* suggests the UGT85C2 enzyme as a rate‐limiting step of SVgly biosynthesis. *UGT76G1* modulates the synthesis of stevioside and rebaudioside A 9, 10. In the present study, the transcription of *UGT74G1* was significantly reduced in polyethylene glycol‐treated plants, which is supported by the results of Yang *et al*. 27, while it is opposed to Hajihashemi *et al*. 16.

It is known that the plant response to drought stress is related to the time and intensity of stress, plant species, genotypes, and environmental conditions 29. The observed variations in the gene transcription of *Stevia* in different experiments can be correlated with different methods to induce drought stress, severity of drought stress, plant growing stages, plant cultivar, harvesting time, etc. Hajihashemi *et al*. 16 induced drought stress in *Stevia* plant by adding 5% polyethylene glycol to MS culture medium while in the present study the gene transcription was studied in plants treated with 10% of polyethylene glycol. Yang *et al*. 27 induced drought stress by stopping watering the pots until the plants wilted. The study of growing stages revealed changes of SVglys contents and the transcription of genes at different growing periods 27. It was observed that moderate water‐deficit stress (8‐days irrigation period) did not significantly affect the SVglys content 30. Accordingly, Karimi *et al*. 31 reported that SVglys content increased when soil moisture was depleted to 60% FC (9‐day irrigation interval), but it significantly decreased under severe drought stress (45% FC). The harvesting time of *Stevia* affected the yield and quality of SVglys content in leaves 32. In conclusion, it can be strongly suggested that the *Stevia* response to drought stress was influenced not only by the intensity of drought stress but also by environmental cues which have a major influence on the transcript levels of genes involved in SVglys biosynthesis and SVglys accumulation in *S. rebaudiana*. However, it should be noticed that *Stevia* cultivation need sufficient irrigation to obtain greater SVglys and profits.

## Conclusion

The response of *S. rebaudiana* Bertoni to different polyethylene glycol treatments outlined in this study confirms the negative effects of polyethylene glycol‐induced drought stress in *Stevia* plant. SVglys content as a critical issue in *S. rebaudiana* was adversely affected by polyethylene glycol treatment which is in a close relationship with the transcription of SVglys biosynthesis genes. These results provide insight into the mechanism by which SVglys accumulation is regulated by gene transcription. In present investigation, the downregulation of *ent‐KS1*,* ent‐KO*,* ent–KAH*,* UGT74G1*,* UGT76G1*, and *UGT85C2* transcription led to a reduction in SVglys content. These findings provided the awareness that severity of drought stress, environmental cues, and methods of treatment resulted in a variation in gene transcription. It indicates that the genes included in the same biosynthesis pathway do not display the same reaction to stress and that this complicates the understanding of the mechanisms. Polyethylene glycol treatment inhibited the *ent‐KO*,* UGT85C2,* and *UGT76G1* in both the present study and our previous study 16. We suggest that the genes involved in SVglys biosynthesis pathway might be classified into two groups of early‐ and late‐induced genes. The three genes, *ent‐KO*,* UGT85C2*, and *UGT76G1*, can be classified as early‐induced genes in response to polyethylene glycol treatment, with the other genes (*ent‐KS1*,* ent–KAH*, and *UGT74G1*) only engaged by increasing the intensity of polyethylene glycol‐induced drought stress and thus classified as late‐induced genes. The three genes of *ent‐KS1*,* ent–KAH*, and *UGT74G1* might be classified as late‐induced genes. Overall, these findings provide new insights into the drought‐response mechanisms in *Stevia* plants. This study on gene transcription in *S. rebaudiana* opens the way for further research to confirm these initial conclusions.

## Author contributions

SH and JMCG conceived, designed, and did the project; SH wrote the paper.
